# The MI bundle: enabling network and structural biology in genome visualization tools

**DOI:** 10.1093/bioinformatics/btv431

**Published:** 2015-07-25

**Authors:** Arnaud Céol, Heiko Müller

**Affiliations:** Center for Genomic Science of IIT@SEMM, Fondazione Istituto Italiano di Tecnologia (IIT), 20139 Milan, Italy

## Abstract

**Summary:** Prioritization of candidate genes emanating from large-scale screens requires integrated analyses at the genomics, molecular, network and structural biology levels. We have extended the Integrated Genome Browser (IGB) to facilitate these tasks. The graphical user interface greatly simplifies building disease networks and zooming in at atomic resolution to identify variations in molecular complexes that may affect molecular interactions in the context of genomic data. All results are summarized in genome tracks and can be visualized and analyzed at the transcript level.

**Availability and implementation:** The MI Bundle is a plugin for the IGB. The plugin, help, video and tutorial are available at http://cru.genomics.iit.it/igbmibundle/ and https://github.com/CRUiit/igb-mi-bundle/wiki. The source code is released under the Apache License, Version 2.

**Contact:** arnaud.ceol@iit.it

**Supplementary information:** Supplementary data are available at *Bioinformatics* online.

## 1 Introduction

Large-scale genomics initiatives aim at systematically cataloguing the complete spectrum of genomic changes in diverse biological and clinical conditions and to elucidate the contribution of those changes to the pathogenesis of disease. These initiatives routinely produce long lists of candidate genes that must be prioritized before more detailed functional studies are warranted. Prioritization of candidates ideally should take advantage of the entire range of available data and produce an integrated picture that permits evaluating the contribution of individual genes to the pathogenic process on a data-driven basis. However, the heterogenic nature of data and bioinformatics tools to manipulate them places numerous hurdles on the way, in particular for biologists with limited bioinformatics skills. We have developed a plug-in for Integrated Genome Browser (IGB) (Nicol *et al.*, 2009) to facilitate candidate prioritization based on network and structural biology criteria in the context of diverse genomic data types that can be loaded as browser tracks. The plugin permits analyzing how genomic variations, in particular missense mutations, may affect the interaction between gene products and other molecules as well as their interference with biological pathways based on the most updated standards and resources available for network and structural biology.

## 2 Molecular networks and structures

**Fig. 1.**
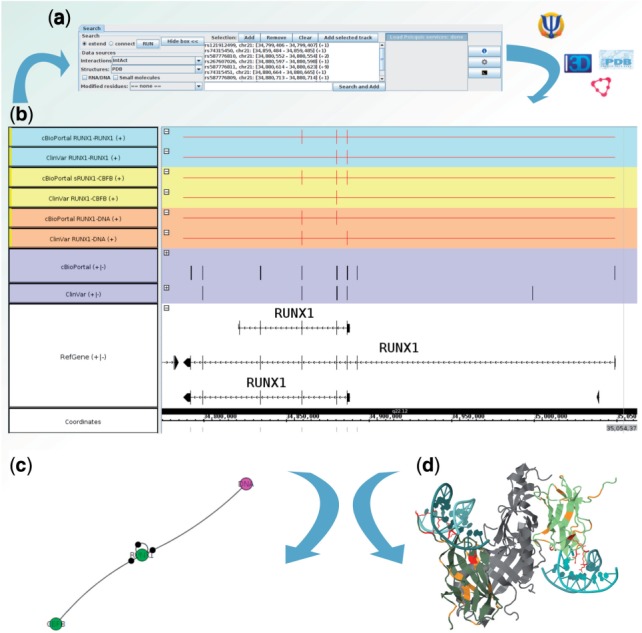
Mapping *RUNX1* variations to molecular interactions. **(a**) Genomic variations for RUNX1 are loaded from ClinVar and cBioPortal (purple tracks). (**b**) Some variations are identified at the interface with CBFB (yellow tracks), DNA (orange track) and RUNX1 (homodimer, blue track). (**c**) Network representation: the black circles on the edges indicate a variation on the interaction interface. (**d**) Structure visualization: affected residues in contact with DNA are displayed in red (PDB:1HD9)

The MI Bundle interrogates on-line interaction databases to identify binding molecules. The query benefits from the adoption of the PSICQUIC (Aranda *et al.*, 2011) standard web service implementation by the major databases. The structures and models for the interactions are obtained either from PDB (Velankar *et al.*, 2011) or Interactome3D (Mosca *et al.*, 2013). PDB provides structures for species and interactions that are not covered by Interactome3D, as well as protein–DNA, protein–RNA and protein–ligand interactions. On the other side, Interactome3D increases the coverage of the network with high quality models. The genomic regions of interest may be associated to one or more transcripts whose sequence is translated and mapped to the Uniprot (Magrane and Consortium, 2011) sequences of the splicing variants, which are later aligned to the associated chains in the PDB files. The atoms of each structure are browsed to extract first the residues encoded by any of the selected genomic regions, and second to identify which of those residues are at the interface between two chains [residues that lose one Å^2^ of available surface area upon binding, calculated with the BioJava library (Prlic *et al.*, 2012)]. Alternatively, it is possible to use the dSysMap database (Mosca et al., 2015), which relies on a pre-defined set of missense mutations. The structures and the contact residues can be selected and displayed in a Jmol frame (The Jmol Team, 2007) (Fig. 1d).

Although the number of available protein structures is considerable, coverage is far from complete (Mosca *et al.*, 2013). Nevertheless, non-structure based molecular interaction networks represent valuable tools for gleaning insight into the complex relationships between genotypes, network properties and phenotypes (Ideker and Sharan, 2008; Vidal *et al.*, 2011). With the MI Bundle, it is indeed possible to build such networks directly from the genomic data and either display them directly from IGB (Fig. 1c) or export them in a standard format that can be analyzed using appropriate software such as Cytoscape (Saito *et al.*, 2012).

## 3 Contact residues and diseases

Recently, Mosca *et al.* (2015) have shown that disease causing mutations are more likely to affect protein protein interaction interfaces. We loaded known genomic variations of *RUNX1* from ClinVar (Landrum *et al.*, 2013), a repository of variations and associated phenotypes, observed principally in patients with acute myelogenous leukemia (Fig. 1a and b). In the MI-Bundle, a new track can be created for each molecular interaction enabling the comparison of the interfaces of a single molecule with different partners at the genomic level. In Figure 1b, we compare the interactions that may be affected by the different variations: two of those (positions K83 and R174) are identified at the interface with DNA only. Previous studies have shown that in the presence of mutation at those sites no DNA binding is observed while the homodimerization capability is preserved (Michaud *et al.*, 2002). Another mutation at position 107 is mapped to the interface with CBFB and may, as suggested by Walker *et al.* (2002), impair this interaction, destabilizing the binding of RUNX1 to DNA and leading to RUNX1 degradation. We loaded additional mutations from cBioPortal (Cerami *et al.*, 2012). Several of those where identified at the interface with RUNX1 (14, of which 10 new), CBFB (10/9) and DNA (5/4), suggesting how those variations may interfere with the molecular network and cause or predispose to disease. Further description of the mapping of RUNX1 variations is available in the Supplementary Material

## 4 Discussion

Based on their observation of the property of disease causing mutations, Mosca *et al.* developed dSysMap, a web server that allows mapping mutations (provided as amino acid positions) to the structures and models of human protein–protein interactions. Structure-PPI (Vázquez *et al.*, 2015) propose a similar strategy that Mechismo (Betts *et al.*, 2014) extends to protein–nucleic acid and protein–ligand interactions and an assessment of the impact of the mutation on the binding properties of the molecules.

The integration of our plugin into a genome browser provides genome biologists access to network and structural analysis: The analyses start from genomic regions, allowing their integration with sequencing pipelines managed by IGB, and to be independent of any preliminary mapping to the protein sequences. The possibility to select the source database extends the range of possible analyses: even when no structures are available, it helps identifying new connections and functional relationships between target genes. Moreover, each new session queries public online databases: each query can be repeated to benefit from new data released in molecular interaction and structure databases.

Finally, the bundle benefits from all the features and future developments of IGB, including the many species available (e.g. Mouse, *A. thaliana* and *E. coli*).

## Supplementary Material

Supplementary Data
