# Supplementary material for: The MI bundle: enabling network and structural biology in genome visualization tools
Source: Bioinformatics. 2015 Jul 25;31(22):3679–81. doi: 10.1093/bioinformatics/btv431 (PMC4817051; doi:10.1093/bioinformatics/btv431)
Supplement: Supplementary Data [file supp_31_22_3679__index.html]

The MI Bundle: Enabling Network and Structural Biology in genome visualization tools. — The MI bundle: enabling network and structural biology in genome visualization tools — The MI bundle: enabling network and structural biology in genome visualization tools — Supplementary Data 

# The MI bundle: enabling network and structural biology in genome visualization tools

## Supplementary Data

files

- Supplementary Data - doc file
