# Supplementary material for: The MI bundle: enabling network and structural biology in genome visualization tools
Source: Bioinformatics. 2015 Jul 25;31(22):3679–81. doi: 10.1093/bioinformatics/btv431 (PMC4817051; doi:10.1093/bioinformatics/btv431)
Supplement: Supplementary Data [file supp_btv431_Supplementary_material.doc]

Supplementary material: mapping sequence variations in gene RUNX1 to molecular interaction interfaces

# Variations from Clinvar:

We downloaded the vcf file for the last human assembly (GCRh38) from <ftp://ftp.ncbi.nlm.nih.gov/pub/clinvar/vcf_GRCh38/clinvar.vcf.gz> (Landru*m et a*l., 2013).

# Integrated Genome Browser:

We installed IGB and the MI bundle as described at <http://cru.genomics.iit.it/igbmibundle/download.xhtml> and loaded the cvf file from ClinVar.

We zoomed into the RUNX1 gene (advanced search tab, RUNX1), selected all variation from ClinVar and ran the MI Bundle with the options:

- Interaction database: Intact
- Structure database: PDB (for accessing DNA/RNA and small molecule data)
- RNA/DNA: yes
- Small molecules: yes
- Modified residues: none

# Browsing the results:

In order to compare visually the contacts involved in different mutations, we can click on the “create button” on the result panel for each interaction for which a structure has been found (green and blue squares in the ”structure column”).

It is possible to personalize the style of the tracks (label an colors) by right clicking on the track label (on the left) and selecting “customize”.

In order to know the position of the contact residues, it is possible either to export the results (text format), or to move the cursor over the position in the respective track. The position is displayed for different possible splicing variants (Figure S1).


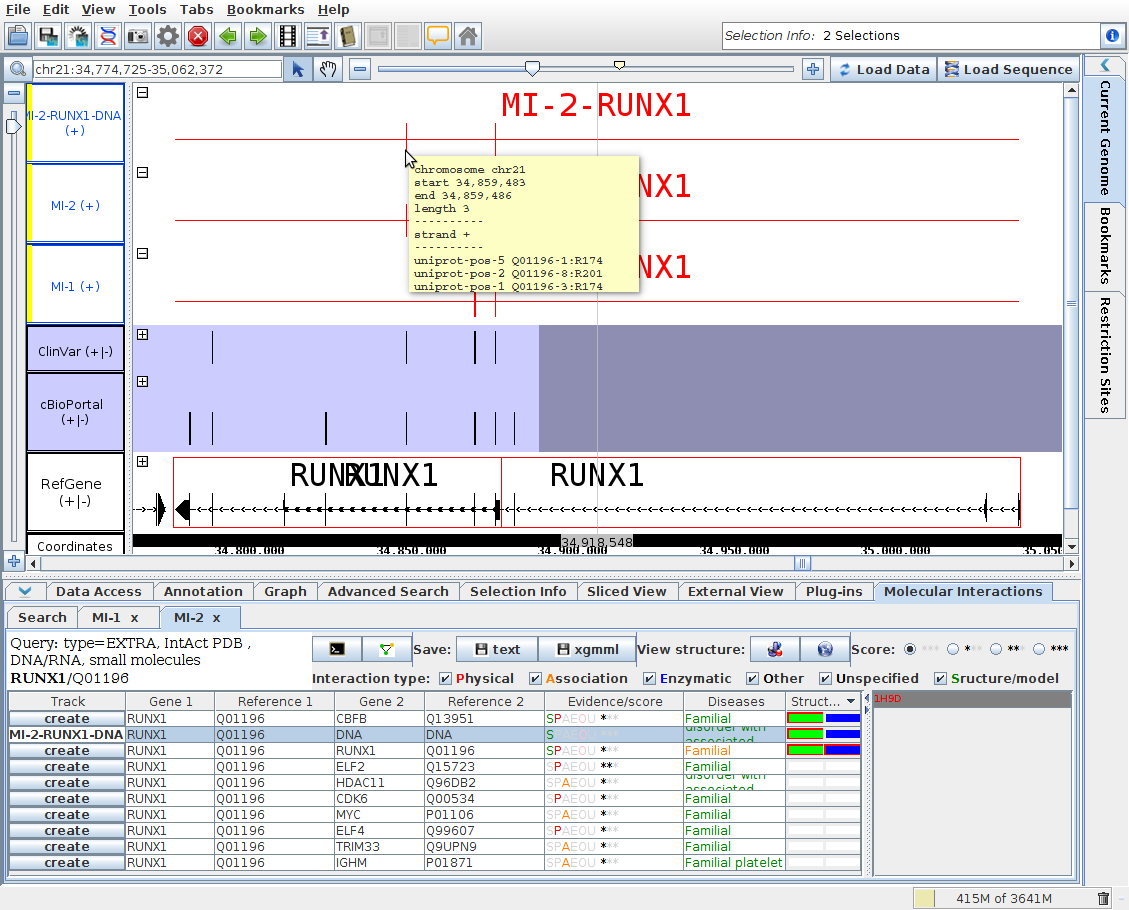


**Fig. S1.**  Browsing the results.

# Variations from cBioPortal:

We selected all missense mutations from the cBioPortal (<http://www.cbioportal.org/>) (Ceram*i et a*l., 2012) for all studies and types of cancer for the gene RUNX1 (tsv format) from which we extracted chromosome, start position and end position. We subtracted 1 from each start position to obtain bed formatted data. We converted the mutation to the GCRh38 human assembly with the liftOver tool (Hinrichs, 2006).

We selected the variations from cBioPortal and ran the plugin again.

# Variations at interface positions:

Table S1 shows the variations of RUNX1 sequence at the interface with DNA, CBFB or RUNX1 (homodimer).

|  |  |  |  | **Contact with:** | | |
| --- | --- | --- | --- | --- | --- | --- |
| **RUNX1 variation** | | **clinvar** | **cbioportal** | **DNA** | **RUNX1** | **CBFB** |
| K | 83 | x |  | x |  |  |
| R | 135 |  | x | x | x |  |
| G | 141 |  | x | x |  |  |
| D | 171 |  | x | x |  | x |
| R | 174 | x | x | x |  |  |
| R | 177 |  | x | x |  |  |
| D | 57 |  | x |  | x |  |
| H | 58 | x | x |  | x |  |
| D | 66 |  | x |  | x | x |
| N | 69 |  | x |  | x | x |
| K | 90 |  | x |  | x |  |
| G | 95 |  | x |  | x | x |
| A | 107 | x | x |  |  | x |
| G | 108 |  | x |  | x | x |
| N | 112 |  | x |  |  | x |
| S | 114 |  | x |  |  | x |
| T | 121 | x |  |  | x |  |
| A | 122 | x | x |  | x |  |
| A | 123 | x |  |  | x |  |
| P | 157 |  | x |  | x | x |
| A | 160 |  | x |  | x | x |
| Y | 162 |  | x |  | x |  |
|  |  |  |  |  |  |  |
|  | ClinVar |  |  |  |  |  |
|  | cBioPortal |  |  |  |  |  |
|  | both |  |  |  |  |  |

**Table. S1.**  Variations at interaction interfaces.

References

Cerami,E. et al. (2012) The cBio Cancer Genomics Portal: An open platform for exploring multidimensional cancer genomics data. Cancer Discov., 2, 401–404.

Hinrichs,A.S. (2006) The UCSC Genome Browser Database: update 2006. Nucleic Acids Res., 34, D590–D598.

Landrum,M.J. et al. (2013) ClinVar: public archive of relationships among sequence variation and human phenotype. Nucleic Acids Res., 42, 980–985.
